# Academy of Stomatology

**Published:** 1896-05

**Authors:** George D. B. Darby

**Affiliations:** Secretary


### ACADEMY OF STOMATOLOGY.

A regular meeting of the Academy was held at its rooms, 1733 Chestnut Street, Philadelphia, January 28, 1896.

After the transaction of the usual routine business Dr. E. S. Talbot, of Chicago, read a paper upon the subject, "Pyorrhœa Alveolaris."

(For Dr. Talbot's paper, see page 203.)

### DISCUSSION.

*Dr. Wm. H. Trueman.*—Certain expressions in the short abstract from the paper given reminded me of a paper written by Dr. Hayden upon pyorrhœa alveolaris in the *American Journal of Dental Science*, and I think it is of interest to refer to it. I think it is page 214, March number, 1822. Dr. Koecker wrote a paper reflecting upon Dr. Hayden, asserting that Dr. Hayden did not understand the nature of the disease. That prompted Dr. Hayden to look the matter up very closely in 1822, and the result of his study was published in the *Medical Recorder* of Philadelphia. He gives, I think, the most exhaustive history of the disease I have ever seen, and, reading it over and reading Dr. Talbot's paper, I am quite sure that the two men may, so to speak, be able to shake hands over a chasm of more than seventy years. I am somewhat inclined to think that both of them have come to the same conclusion.

Dr. Talbot states that the disease is increasing. I think that would be the sentiment of every dentist who has been two score years in successful practice; it may be that when a dentist has many patients with the disease, it gives him the impression that it is increasing. I do not think so; neither do I think it is due to dental operations, from the fact that all the old writers speak of it as very prevalent and very destructive to the teeth, so much so that they termed it devastation of the teeth, and a hundred years ago that expression was about the same as is now applied to pyorrhœa alveolaris.

It is a question with me, in many cases, where the pathological condition begins. In early life sometimes the gum-margins are receding, perfectly healthy in color, and this continues to such an extent that the teeth become loose, and then results a pathological condition due to the loosening of the teeth. I have found that where the teeth were mechanically held firm the pathological conditions subside.

In regard to the gouty diathesis, I have not seen anything to lead me to think it has anything to do with it. I have seen conditions of the mouth indicating strongly marked pyorrhœa, and no other symptoms of uric acid diathesis; in many cases I have put patients under lithia treatment, without noticing any benefit at all. In several cases where patients lead a very sedentary life I have urged a change, and it brought a very marked improvement.

With regard to the cure of the disease, I think there is but one, and that is the forceps. I have never seen a case that has been cured.

As long as the treatment is continued there is improvement; if the treatment is discontinued the disease returns. I have observed that if the teeth are held firm, it aids in effecting a cure. I have tied them with a ligature in many cases, with great improvement, and in a number of cases recently, probably eight or ten, I have drilled holes through them and put in a bar and held them perfectly firm.

I had a case some time ago that was cured spontaneously, without any treatment whatever. A patient had been coming to me for some time, a man about thirty years of age, and finally the trouble reached such a stage that I told him I could do nothing for it, and if any one could, to let him have the opportunity; the teeth were loose above and below, the pus was exuding, and the mouth was in a very unhealthy condition. He had always taken good care of his teeth. I saw him again in a year, and there was the greatest change, and with very little recession of the gums. He had, in the mean time, made no change in his mode of living, and had not been under medical treatment.

There are some cases where, under this condition, the teeth are removed and the roots are perfectly smooth, and in others absorption has gone on, leaving them like needle-points. I would like to know the cause.

*Dr. W. G. A. Bonwill.*—I read Dr. Talbot's paper before I came here. You all know my treatment in regard to pyorrhœa. Dr. Talbot's paper reaches some points very clearly. I cannot agree with him so far as his treatment is concerned. It is time some of us had learned to treat pyorrhœa. Of the number of teeth he had extracted I find in his essay that fifty per cent. come from his own office. I should judge from this that he had not much success in the treatment of his cases. I can agree with him in one thing, so far as I believe and have seen, and that is his view of the hypothesis in regard to gouty diathesis. I never had occasion to see the etiology of the disease in that light. I do not agree with him that it commences on the gums on the alveolar border, nor that the deposit should be left there. That is a very ultra measure. Even he, I judge, would not like us to think he advocated that. I would not like to think that any man would do it, nor do I care to see a man with good sense recommending anything of the kind.

I agree with him that bad dentistry has done more than almost any other one thing in producing this disease. It is not necessary to state in what way, but he does not tell us what is good dentistry, nor how to cure it by good dentistry. I have no doubt these cases can be cured.

I am a little surprised at the doctor using iodine. In my practice the use of it is almost obsolete.

There are a good many points that may be taken up, but the whole thing has been discussed over and over again, and nobody seems to come down to the fact that pyorrhœa can be cured. I feel that he leaves us just where he found us.

I treat pyorrhœal pockets as fistulas; when you have once done it let nature alone. I treat the case as a surgical one, burn it out. Do your work right, and nature does the balance.

I had a case sent to me by a dentist in Philadelphia. He told the patient he could do no more. Really, there was no trouble in the case, but the dentist had kept him running to his office constantly. He dabbled in it so often, nature had no chance to make a cure. In three treatments, two hours the first time, I went right through the whole thing, burned it out as if I were burning out a cancer; then let him come back in a few weeks; and as there was no further trouble I sent the gentleman back to his dentist with my compliments.

At some future time I shall write up what I have to say on this subject.

*Dr. D. D. Smith.*—There are points in connection with this subject which have never been considered, so far as I know, or, if they have, there has not been sufficient emphasis placed upon them. Dr. Talbot touched on some of them to-night, and what he said agreed mainly with my experience in connection with the disease.

In the first place, there is the age of the patient to be taken into consideration. We talk of this disease as if it were, like decay, finding expression in every mouth, in the mouth of the young subject, and in the temporary teeth. Often I have heard it declared it has been found in the temporary teeth, and also in old age.

In my experience it is confined absolutely to middle life, just at the age where we do not want to find it. We do not find it in young subjects. Dr. Talbot's paper speaks of the disease being found in connection with a person fifteen years of age. In a practice of thirty-three years I have failed to find it in a patient as young as this. I do not remember ever seeing it in a case of twenty years of age; possibly I may have. Persons who give expression to this disease are people well along in years,—from twenty-five to forty years.

Another point I failed to notice in his paper is, I have no hesitation in saying, that it is always found in connection with good teeth; teeth exempt from decay. He speaks of the teeth coming out smooth without any deposit on the roots. So they do. We find just that condition of things. But what sort of teeth are they? they are teeth that have never decayed or have ceased to decay; they are teeth without an expression of decay about them. I have never found it associated with poor teeth and teeth liable to decay.

If this is the case it cannot be from uric acid poisoning. It cannot be from irritation of the gums from a local irritant, or a constitutional irritant which expresses itself at the margin of the gum. There must be something else associated with it.

How does this disease attack the teeth? From what we hear from the papers and discussions, we might suppose the disease attacked the teeth anywhere on the roots. Where does it appear? Between the teeth. What else do the teeth do? They turn and twist in their sockets as if there was some irresistible force expelling them outward or inward. There is a force at work on the roots of the teeth which is actually twisting them out of their alveolar sockets. What is it? What does it? Why is it always associated with good teeth? You have all seen a central incisor attacked. Where? On the mesial portion of the root, twisting that tooth and rotating it in its socket, and you will find in all these cases that there is an opening between the two teeth; in every case a space between the two. The teeth are forced in the direction of the least resistance, whatever that may be. If it is a lateral it is almost always attacked on the distal face, and if it is a central it is almost universally attacked on its mesial face and forced backward. What occasions this? Here in the paper we see nothing of this particular expression of the disease in connection with strong, sound teeth.

In regard to tobacco I have this to say, that while its use is an exceedingly filthy habit, I have never seen any ill effects from chewing tobacco except wearing down the teeth. I frequently recommend my young patients to chew gum. I think it is one of the best things to make good teeth. The essayist speaks about rubber dam being one of the exciting causes. I cannot agree with him. I do not believe rubber dam or regulating appliances ever did such a thing in the world as to excite pyorrhœa. They are almost always in connection with children. I have never found it in connection with young people. I cut off these pyorrhœal teeth and put on bridge-work on purpose to save them, and do it effectually. As to pregnancy, why should it be an exciting cause? Pregnancy is often the cause of decay of the teeth. We always look for it and expect to find it.

While I agree with Dr. Talbot in many respects, I do not agree with him as to the exciting causes. I think the root of the trouble lies in the dental pulp. Investigation on the subject will go to show that the dental pulp is at fault in connection with pyorrhœa, where it exists in its true form, in the solid teeth of mature life. It has made the tooth too solid, it has made it of too good material, and the result is that the pericemental membrane becomes irritated and begins to withdraw itself; and it is just like any other irritant anywhere else, and nature begins to throw it out, and begins at the weakest point. That is why the twisting and turning occurs. What proof have we of this? I do not say it is all the dental pulp, but I believe the dental pulp is at fault. It has overdone its work, it has made the tooth itself a local irritant, and nature is making an effort to throw it out.

In the case of a good, healthy tooth with a devitalized pulp, you will never find pyorrhœa attacking it, no matter even in a mouth where pyorrhœa is running a full and free course; the devitalized tooth will be exempt. Why? Simply because the cemental structure about the root of the tooth is in its normal condition. There is nothing to interfere with it, and it remains there for the support of that tooth.

I cannot fight pyorrhœa as Dr. Bonwill does, burn it out and let it go; it will not do it for me. In one case I treated a patient for a number of years, and finally drilled into the pulp and destroyed it. That did not bring the tooth back. It remained in that twisted position, and finally I cut it off and put on a crown, and it will take a dentist to know there has been anything done in that mouth. The disease seems to be cured.

*Dr. James Truman.*—I hardly feel like expressing a word, and I would not do so were it not for the fact that my friend, Dr. Talbot, has come here with a paper that I generally indorse.

It is a gratification to me to listen to it, because I regard it as thoroughly scientific. He has searched for the truth through proper channels. The clinical experiments he made in Chicago confirm my own views that the uric acid theory never had any firm basis as a theory of the etiology of pyorrhœa.

There has been much said on the subject here in Philadelphia. We have all written and spoken on it, and we never will agree. I do not even agree with Dr. Talbot exactly that it begins in the gum-tissue. I fully agree with him that the deposits really have very little to do with it; in fact, you can treat a case of pyorrhœa without removing the deposits. I know that is radical. I am a little surprised to hear Dr. Talbot take that position, but I believe it to be true; that the deposits are simply secondary, and that it has little to do with the progress of the disease. The disease is primarily caused by micro-organisms. That, I think, I settled to my own satisfaction twenty-five years ago in microscopical examinations, and I have never seen any reason to change it. I know very well that in the treatment of this disease you must first attack those pathogenic germs which exist around the gingival border if you expect to have success, and it is astonishing to me to hear a man, who has been in practice as long as my friend William Trueman has, say that nothing but the forceps will relieve this trouble. Where are we? What are we here for as dentists if we cannot do any better than that? Now, as far as I am concerned, I must say that each case can be treated and restored to health, but it must be treated right. I have a tooth in my own mouth which I treated twenty-five years ago for pyorrhœa, and it is there yet, and I know it will remain there as long, probably, as I remain on this planet.

It is an error that it does not appear except in middle life; I have treated some very bad cases at twenty years of age, and successfully.

We will never come to any agreement until we come to a clear understanding of the etiology of the whole matter. I am satisfied with the treatment I adopted, and I gave it to the profession long ago.

*Dr. E. T. Darby.*—I have listened to Dr. Talbot's paper, and I think he is right, and when I hear Dr. Peirce, I think he is right, and I think they are both right. I do not believe all cases of pyorrhœa are from the same cause. I believe most fully that some cases have their origin below the gingivæ and express themselves in little tumors along the gum-border. That is the incipiency of the disease. I have thought, at times, the inflammation was the result of the density of the roots. I have seen other cases of pyorrhœa such as Dr. Talbot describes when I thought the origin of the disease was at the gingivæ. Therefore I say all cases of pyorrhœa do not start from the same cause.

As to the cure, I occupy a middle position. I believe pyorrhœa, before it has progressed too far, can be cured, substantially, but all cases of pyorrhœa that have come to my hands have required subsequent and constant treatment. I do not think it can be cured in one or ten sittings, either at intervals of six or ten months or ten years. It requires constant and careful attention. But I should be sorry to believe the forceps to be the only remedy we have for many of the cases that come to us.

I cannot agree with Dr. Smith that the disease always arises necessarily on the mesial surface. I have frequently noticed it beginning at the palatal surface. Neither can I agree with him that it is always on the approximal surface in regard to the posterior teeth. I have seen the disease start upon the palatal root more frequently than elsewhere, and that root become perhaps entirely denuded before the disease manifested itself elsewhere around the gingivæ.

*Dr. H. H. Burchard.*—A full and adequate discussion of Dr. Talbot's paper would be more lengthy than the essay itself, as it involves the long list of disorders known to produce the condition called pyorrhœa alveolaris. I notice one point in his investigations which I had marked out for my next summer's work,—the influence of tabes dorsalis upon disorders of the teeth. I was, however, and am, prepared to look for more extensive trophic changes due to tabes. It would be curious, indeed, were the fifth cranial nerve to be exempt from degenerative changes in a disorder which involves many of the others. When we have an opportunity to read Dr. Talbot's paper in print and to deliberately examine his data I believe we will find he has contributed some valuable material to the subject of the pathology of gout.

Hearing the discussion of one of the early papers of Professor Peirce upon this subject, it became evident that some of his critics did not pay sufficient attention to the clinical history and morbid anatomy described by him, in which he clearly differentiated between two diseases; the first a pyorrhœa beginning as a marginal gingivitis and accompanied by the deposit of dark, scaly, and hard bodies beneath the gum-margin, this being the starting-point of an inflammatory degeneration of pericementum which eventually caused the loss of the tooth; the second class those which he described as accompanying and due to the gouty diathesis, these having the incipiency of the dental disease at some portion of the pericementum beyond the gum border. I have seen and recorded such cases, one where there was a well-defined calculus formed upon the upper, the apical portion of the pericementum, the membrane beyond the deposit towards the neck of the tooth being intact.

The next case was one in which there was no inflammatory disturbance of the gingivæ, but half-way towards the apices of the roots of two teeth were swellings, inflammatory tumors, which, when incised, showed the roots of the teeth to be denuded of the pericementum and a partial loss of the alveolar walls; these two cases are cited as being distinctive examples of correspondence with the morbid joint anatomy of other parts found accompanying gout. That all persons who are the subjects of the gouty diathesis are not the victims of pyorrhœa or phagedenic pericementitis is no sufficient argument against this special constitutional condition being a predisposing and exciting cause of the dental disease. Many patients who are evidently and markedly gouty never have any inflammatory disturbance of the metatarso-phalangeal articulation. It exhibits itself in the retaining structures, the articulative tissues of the teeth, because this happens to be the weak joint, a *locus minoris resistentiæ*. Why this is a weak joint I have endeavored to explain in papers written for the *Dental Cosmos* and for the Philadelphia County Medical Society, and have as yet found no reason to alter the opinions advanced in those essays; in fact, time and other essays upon this matter confirm the hypothesis advanced.

In regard to the small percentage of cases exhibiting the murexid reaction, Dr. Kirk and myself noted this soon after the publication of Professor Peirce's essay, finding many of the deposits giving alone the reaction of calcium phosphate; however, several of these cases were those of patients markedly gouty, having a family and personal history of gout.

It is unquestionably true that deposits occur upon the lateral aspects of the roots of teeth without detachment of the gum from the necks of the teeth; secondly, it is equally true that inflammatory disturbance and abscess may occur in similar situations, without the pulp of the tooth being dead; several such cases are recorded in the proceedings of this academy.

Teeth may be lost through the progressive molecular loss of their retentive apparatus and not have their roots the seat of deposits. Many cases are recorded which resisted purely local treatment and yet were clearly benefited through the patients' adherence to an anti-gout regimen and by anti-gout therapeusis. As to the pathology involved, I have believed for the past two years the following to furnish a rational explanation. The pericementum, for reasons which I advanced nearly two years ago, becomes in some persons a weak articulative tissue. The blood-vessels supplying it are, owing to causes which underlie the gouty condition itself, the seat of inflammation; through the swelling of the tunica media the lumen of a vessel or of vessels become obstructed, and there is a death of cellular elements dependent upon that vessel for nutrition,—that is, a greater or less area of pericementum dies.

In this area, whether due to some special selective affinity or to an acid reaction of the dead tissues, a deposit of urates occurs. This may receive fresh accretions through subsequent attacks, and, during its presence, a greater or less degree of irritation is kept up and inflammatory degeneration of the tissues about it occurs.

This corresponds closely with the history of a gouty affection in any other part.

For a solution of the pathology involved in the loss of teeth from deposits which are first found just beneath the gum-margin we need look for no constitutional condition as explanatory; local causes furnish a perfectly rational explanation for it; however, I have seen many of these cases which occurred in gouty patients. Given lactic fermentation, which is present in all mouths perhaps, and coagula are formed by the action of dilute lactic acid upon the mucin of the several secretions about the mouth, and the lime-salts of the saliva are precipitated and entangled in the coagulum, and in any spot where these particles may find lodgement there is a nidus about which will form calculus. Beneath the margin of the gum they act as irritants, the mucous glands have an altered secretion and this reacts upon the soft deposits, altering their chemical and physical structures, a gingivitis supervenes, the gum-tissue swells and is detached from the neck of the tooth, and micro-organisms have a pocket in which to develop. It is not unlikely that the streptococci invading such areas may give rise to phagedæna. In any event the pathology differs from that of the loss of teeth by the gradual encroachment of salivary calculus only in anatomical situation and not in the essence of the process. There is one point in regard to this matter which needs an emphasis, which I endeavored to place upon it in an article in the INTERNATIONAL DENTAL JOURNAL last year, and that is, even though the case be gouty, it is possible that, by the perfect removal of deposits, the securing of surgical rest, and local asepsis, such cases may be cured for the time being, but there is no assurance that the disease will not reappear with the pathological conditions which precede an attack of gout.

No doubt we all agree with Dr. Younger in this matter, that the essential thing is to get rid of the deposits. I have written somewhat extensively upon this subject and have made careful examination of many cases, as to clinical and family history, and I am more and more inclined to adhere to the doctrines I have already advanced, which were promoted mainly by the teachings of Dr. Peirce and later by a paper of Dr. Reese; this essay, however, antedated that of Dr. Peirce's by several years.

In regard to Dr. Talbot's asserted belief that the deposits found in the general class of disorders are unirritating, characterized by pyorrhœa alveolaris, I do not think he will have the concurrent opinion of clinicians in general. His analogy of the exostoses does not hold; the calcic salts deposited in this hypertrophic condition are probably in the form of calcoglobulin; those deposited as precipitates from chemical solution are physically irritating in themselves; besides, even an exostosis is due to irritation, and clinical evidences show it perpetuates the irritation.

*Dr. Talbot.*—There is one point in regard to pyorrhœa advanced by Dr. Peirce that I do not understand. I cannot understand how, if the disease is constitutional, the deposit will take place on one tooth and on one side of a tooth, and not on the other. I cannot understand why these men who advocate constitutional treatment are so positive in their local treatment. If it is constitutional, why simply removing it with an instrument and treating it locally for a time? Why that is all that is necessary? If it is constitutional, it must be treated constitutionally. Simply removing the calcic deposit from the root of the tooth does not cure the disease if it is constitutional. That certainly knocks that theory all to pieces.

In regard to the use of iodine, Dr. Bonwill wants to know when I would stop. I know by each particular case when to stop. I did not advocate it in a medical way. With iodine you can make the gum tight around the root, and the pus-germs cannot get in there to start up the trouble again.

In regard to hard teeth, spoken of by Dr. Smith, there is no doubt but that persons with such teeth suffer most from this disease. He says the pulp has something to do with it. The pulp has nothing to do with it. The pulp recedes. The tubuli of the dentine are filled in with secondary material, and the tissues are harder. What becomes of the pericemental membrane? It has lost its office. The membrane has lost its function. Is that reasonable?

When I come to a meeting I try to relate facts; there is no guesswork about it. I state what I know. I thank you for your kindness, and am much obliged to you for your discussion.

Dr. James Truman moved that the thanks of the society be voted to Dr. Talbot. Carried.

Meeting adjourned.

George D. B. Darby,
*Secretary.*

